# Chloroform—a Substitute for Ether

**Published:** 1847-12

**Authors:** 


7. *Chloroform—A Substitute for Ether.*—To the Editor of the Boston Medical and Surgical Journal:—Dear Sir,—Prof. Simpson, of Edinburgh, has lately published a pamphlet upon the use of chloroform as a substitute for ether, in preventing the pain of surgical operations. A friend sent me a copy the day it was published, and I have experimented with the article before the students of the Baltimore Dental College. Although the tests have not been complete, as indeed they could not be in the few trials of a new agent in the use of which it was necessary to acquire dexterity and exercise prudence, I am satisfied that chloroform is much superior to ether, and not liable to similar objections. I send, with this, a proof sheet of the American Journal of Dental Science, containing Dr. Simpson's account of this new agent.

Very respectfully yours.

*Baltimore, Dec.* 19, 1847. C. A. HARRIS.

P.S.—Since the above was written, I have had another opportunity of testing the anæsthetic properties of chloroform in a very difficult operation, and with complete success. C. H. A.

[The sheet alluded to above has been received. We have space this week only to say, that chloroform is the perchloride of formyle—formyle being, in chemical language, the hypothetical radical of formic acid, which latter is obtained from the red ant, and also latterly from sugar, starch, and indeed most other vegetable substances.—*Bost. Med. and Surgical Journal.*]

